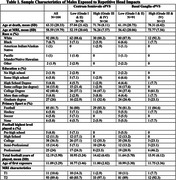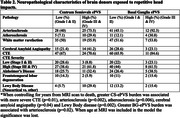# Risk factor, neuropathological, and clinical correlates of perivascular spaces in brain donors exposed to repetitive head impacts

**DOI:** 10.1002/alz.085699

**Published:** 2025-01-09

**Authors:** Sydney Mosaheb, Frances Rodriguez, Bobak Abdolmohammadi, Madeline Uretsky, Christopher Nowinski, Daniel H Daneshvar, Yorghos Tripodis, Brett Martin, Joseph N. Palmisano, Chad Farris, Bertrand Russell Huber, Thor D. Stein, Ann C. McKee, Jesse Mez, Jose Rafael Romero, Michael L. Alosco

**Affiliations:** ^1^ Boston University Chobanian & Avedisian School of Medicine, Boston, MA USA; ^2^ Concussion Legacy Foundation, Boston, MA USA; ^3^ Harvard University School of Medicine, Cambridge, MA USA; ^4^ Boston University School of Public Health, Boston, MA USA; ^5^ Boston University Alzheimer’s Disease Research Center, Boston, MA USA; ^6^ Boston Medical Center, Boston, MA USA; ^7^ Boston University Chronic Traumatic Encephalopathy Center, Boston University Chobanian & Avedisian School of Medicine, Boston, MA USA; ^8^ VA Boston Healthcare System, Boston, MA USA; ^9^ VA Bedford Healthcare System, Bedford, MA USA; ^10^ The Framingham Heart Study, Framingham, MA USA

## Abstract

**Background:**

Enlarged perivascular spaces (ePVS) on MRI can signal impaired cerebral fluid clearance and predict dementia risk. Risk factors and biological correlates of ePVS are uncertain partially due to the lack of pathological correlation studies. Repetitive head impacts (RHI) from contact sports might represent one risk factor for ePVS, given their association with vascular pathologies and chronic traumatic encephalopathy (CTE), a neurodegenerative disease characterized by perivascular p‐tau aggregates. We examined risk factors, neuropathological, and clinical correlates of antemortem MRI ePVS among brain donors exposed to RHI.

**Method:**

The sample included 104 brain donors exposed to RHI from the UNITE brain bank. Clinical MRIs were obtained through medical record requests. A stroke neurologist used established visual rating scales (0=no ePVS, 4= >40 ePVS) to rate ePVS in the centrum semiovale (CS‐ePVS) and basal ganglia (BG‐ePVS) on axial T2 (n=11, 10.6%) or T1 (n=93, 89%). ePVS were coded as low (1/2) or high burden (3/4). Neuropathological diagnoses were made using established criteria. Years of football served as a proxy for duration of RHI. Antemortem dementia diagnoses were made through consensus conferences. Regression‐based analyses tested the association between ePVS with years of football play, various neuropathologies, dementia status and FAQ. Analyses controlled for years from MRI scan to death.

**Result:**

Sample characteristics are in Table 1. The most common pathological diagnosis was CTE (n=71, 68%). 34 (32.7%) and 13 (12.5%) had high CS‐ePVS and BG‐ePVS burden, respectively. More years of football play was associated with greater CS‐ (OR=1.12, 95% CI=1.03‐1.22, p=0.01) and BG‐ePVS (OR=1.12, 95 CI=1.01‐1.24, p=0.03). Effect sizes remained when age at MRI was included for both CS‐ and BG‐ePVS (OR=1.09) but statistical significance was diminished. Greater CS‐ePVS was associated with more severe CTE, arteriosclerosis, atherosclerosis, cerebral amyloid angiopathy and Lewy body disease (Table 2). BG‐ePVS was only associated with arteriosclerosis. Greater CS‐ePVS were associated with increased odds for having dementia diagnosis (OR=5.41, 95% CI=1.42‐20.54, p=0.01). Clinical and pathological correlations were not statistically significant when age at MRI was included.

**Conclusion:**

Enlarged PVS might be long‐term MRI consequences of exposure to RHI, but their age independent pathological and clinical correlates in this setting remain uncertain.